# A multiverse of α-synuclein: investigation of prion strain properties with carboxyl-terminal truncation specific antibodies in animal models

**DOI:** 10.1186/s40478-024-01805-z

**Published:** 2024-06-10

**Authors:** Grace M. Lloyd, Stephan Quintin, Zachary A. Sorrentino, Kimberly-Marie M. Gorion, Brach M. Bell, Brooke Long, Giavanna Paterno, Benoit I. Giasson

**Affiliations:** 1 Department of Neuroscience, College of Medicine, University of Florida, BMS J483/CTRND, 1275 Center Drive, Gainesville, FL 32610 USA; 2 Center for Translational Research in Neurodegenerative Disease, College of Medicine, University of Florida, Gainesville, FL 32610 USA; 3 McKnight Brain Institute, College of Medicine, University of Florida, Gainesville, FL 32610 USA

**Keywords:** α-Synuclein, C-terminal truncation, Prion-type, Strains, Parkinson’s disease, Multiple system atrophy, Synucleinopathies, Astrocytes, Microglia

## Abstract

Synucleinopathies are a group of neurodegenerative disorders characterized by the presence of misfolded α-Synuclein (αSyn) in the brain. These conditions manifest with diverse clinical and pathophysiological characteristics. This disease diversity is hypothesized to be driven by αSyn strains with differing biophysical properties, potentially influencing prion-type propagation and consequentially the progression of illness. Previously, we investigated this hypothesis by injecting brain lysate (seeds) from deceased individuals with various synucleinopathies or human recombinant αSyn preformed fibrils (PFFs) into transgenic mice overexpressing either wild type or A53T human αSyn. In the studies herein, we expanded on these experiments, utilizing a panel of antibodies specific for the major carboxyl-terminally truncated forms of αSyn (αSynΔC). These modified forms of αSyn are found enriched in human disease brains to inform on potential strain-specific proteolytic patterns. With monoclonal antibodies specific for human αSyn cleaved at residues 103, 114, 122, 125, and 129, we demonstrate that multiple system atrophy (MSA) seeds and PFFs induce differing neuroanatomical spread of αSyn pathology associated with host specific profiles. Overall, αSyn cleaved at residue 103 was most widely present in the induced pathological inclusions. Furthermore, αSynΔC-positive inclusions were present in astrocytes, but more frequently in activated microglia, with patterns dependent on host and inoculum. These findings support the hypothesis that synucleinopathy heterogeneity might stem from αSyn strains with unique biochemical properties that include proteolytic processing, which could result in dominant strain properties.

**Supplementary Information:**

The online version contains supplementary material available at 10.1186/s40478-024-01805-z.

## Introduction

Synucleinopathies are a group of neurodegenerative disorders that includes Lewy body diseases (LBD) such as Parkinson’s disease, and non-Lewy body diseases such as multiple system atrophy (MSA), defined by the presence and type of pathological α-Synuclein (αSyn) inclusions in the central nervous system (CNS) [9, 12]. The prominent neuronal αSyn inclusions in LBD are referred to as Lewy bodies and Lewy neurites [12, 46]. In MSA, αSyn inclusions predominantly form in oligodendroglia and are known as glial cytoplasmic inclusions (GCIs) [9, 12]. Synucleinopathies are further distinguished by their clinical presentations, where LBD can present as Parkinson’s disease (PD) and dementia with Lewy bodies (DLB), whilst MSA may present with differing Parkinsonian (MSA-p) or cerebellar symptoms (MSA-c) [8, 25, 41].

The ability of αSyn to produce divergent pathologies has led to the adoption of the strain hypotheses akin to the role of prion protein in human prion diseases, where distinct conformations of misfolded protein lead to varied disease phenotypes [37]. Similarly, αSyn pathology has been shown to propagate in a disease-dependent manner and this variability in the distribution of αSyn pathology amongst synucleinopathies may be due to intrinsic features of different strain-like forms of misfolded αSyn [2, 21, 22]. Congruent with this notion, brain extracts from MSA cases display enhanced seeding potency compared to other synucleinopathies [2, 21, 32, 40]. Additionally, aggregated αSyn from MSA brains are resistant to proteolytic digest compared to LBD [31]. Furthermore, studies have shown that the structural motifs and proteolytic digest patterns which differentiate disease-derived strains can be conferred onto seeded recombinant αSyn [24, 26, 40]. In a previous study, we distinguished the seeding properties of brain lysates from cases of MSA, dementia with Lewy Bodies (DLB), Alzheimer’s disease with amygdala predominant Lewy bodies (AD/ALB), and preformed recombinant αSyn fibrils (PFFs) in their ability to induce phosphorylated Ser129 (pS129) positive αSyn pathology in experimental mouse models [21]. Consistent with other studies, MSA derived seeds were far more efficacious in inducing pS129 αSyn pathology throughout the neuroaxis compared to lysates generated from DLB and AD/ALB, which resulted in limited αSyn pathology predominately near the brain inoculation site [21].

αSyn pSer129 is a common post-translational modification (PTM) found in Lewy bodies and GCIs and therefore is extensively used for histologic detection of αSyn inclusion pathology [3, 46]. However, pathological αSyn carries an array of other PTMs including ubiquitination, nitration, O-GlcNAcylation, and proteolytic truncations [3, 39]. Among these PTMs, carboxyl-terminally truncated αSyn (αSynΔC) has been a significant area of study as the carboxyl (C)-terminus of αSyn is involved in maintaining its very high solubility property, whilst physiological cleavages in this domain accelerates its aggregation into misfolded amyloidogenic forms [33, 43]. In addition to presenting enhanced aggregation, recombinant αSynΔC fibrils display an alternative microscopic structure with more “twists” and increased resistance to proteolytic digestion, reminiscent of MSA derived fibrils [16]. Specific in vitro proteolytic assays have been used to differentiate disease strains, as alternative digestion patterns indicate the discrete molecular structures that may exist between strains [11, 31, 40]. The transmissible structural and biochemical features mediated by αSynΔC may offer insight into the PTMs involvement in generating the structural polymorphisms of αSyn disease strains.

In light of converging mechanisms surrounding αSynΔC, we have developed a panel of highly specific antibodies to αSynΔC (x-103, x-114, x-115, x-119, x-122, x-125 and x-129) which we previously used to characterize pathology in human cases of PD, DLB, AD/ALB and MSA [14, 33]. We have also characterized the formation of specific αSynΔC (x-103 and x-114) as an early event in the endo/lysosomal processing of PFFs in cultured cells [33]. Our recent study of the time-dependent formation of αSynΔC in intramuscular (IM) PFF seeded mice expressing familial PD mutant A53T human αSyn (TgM83^+/-^) revealed that various αSynΔC occur early in parallel with pS129 pathology, however, the prevalence of αSynΔC species varied depending on the specific C-truncation and the neuroanatomical regions studied [20]. Given that αSynΔC is highly prevalent in diseased human tissues and may mediate features reminiscent of pathological strains, in the present study we assessed the αSynΔC profile in two transgenic mouse models seeded with MSA brain lysate or recombinant PFFs [21].

## Materials and methods

### Mouse lines and intrahippocampal stereotactic injection

**Fig. 1:**
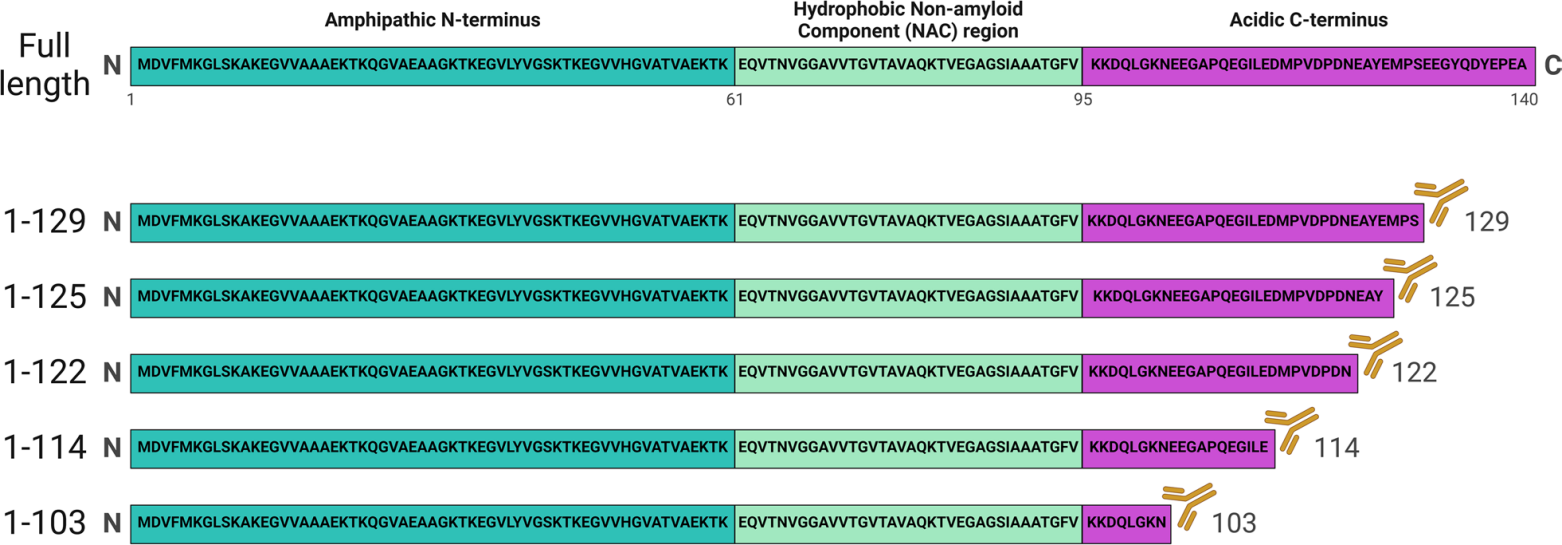
Illustration depicting the epitopes for the αSynΔC specific antibodies. Comparison of the primary sequence and structural domains of the full-length human αSyn protein with the C-terminally truncated variants recognized by the antibodies employed in this study

The investigations conducted here leverage our previously published research on prion-type inclusion αSyn pathology in human αSyn transgenic mice [21] and a series of antibodies specific for αSyn cleaved at specific residues in the C-terminal region (Fig. 1). The breeding, housing and experimental procedures were detailed in our earlier work [21]. To briefly summarize, we examined the CNS tissue of two types of transgenic mice: (Prnp-SNCA*A53T)^+/-^, referred to as TgM83^+/-^, and (Prnp-SNCA)^+/-^ , referred to as TgM20^+/-^ [7]. The mice were housed in a controlled environment with a 12-h light/dark cycle and had ad libitum access to food and water. Both male and female mice were included in the study, and all animal experimental procedures were conducted with approval and compliance with the regulatory policies of the University of Florida Institutional Animal Care and Use Committee.

The tissue [21] analyzed in this study originated from mice that received hippocampal injection of either human brain lysate sourced from two individuals with MSA (see Table 1) or human recombinant αSyn PFFs. As outlined in Table 2, a total of 26 mice was investigated in the current study. As described in the a forementioned study, animals were euthanized either at the predetermined endpoint of 6 months post-surgery or upon the onset of fatal motor symptoms at about 4 months post-surgery for TgM83^+/-^ mice [21].

**Table 1:** Human cases used to generate inoculums

Key resources table: MSA cases								
Patient designation	Neurological diagnosis	Primary pathological diagnosis	Secondary pathological diagnosis	Thal	Braak	CERAD	Age	Sex
MSA-1	MSA-P	MSA	PART	0	II	none	77	M
MSA-2	MSA-C	MSA	AD low; CAA	3	I	sparse	71	M

**Table 2:** Mice used in the studies

Inoculum	n	Injection site	Approximate time post seed injection
TgM20^+/-^ *(Tg (Prnp-SNCA)*^+/-^*)*			
Cerebellum insoluble MSA 1	3	Right Hippocampus	6 months
Cerebellum insoluble MSA 2	4	Right Hippocampus	6 months
4 µg human PFFs	5	Right Hippocampus	6 months
TgM83^+/-^ (Tg (Prnp-SNCA*A53T)^+/-^*)*			
Cerebellum insoluble MSA 1	5	Right Hippocampus	4 months
Cerebellum insoluble MSA 2	5	Right Hippocampus	4 months
4 µg human PFFs	4	Right Hippocampus	4 months
Total	26		

### Antibodies

Mouse αSyn monoclonal antibodies used include those specific for human αSyn C-terminally truncated at residues 103 (2G5), 114 (1A2), 122 (10A4), 125 (5C1) or 129 (2G7) [14, 33], and antibody 3H19 targeted to residues 110–119 within αSyn [21]. Other antibodies include rabbit anti-CD11b (Abcam) and rabbit anti-GFAP (DAKO).

### Tissue processing and histopathological analysis

Methods for mouse tissue fixation and processing were previously described [21]. The methods for immunohistochemical analysis was performed as previously described [20] and summarized in Table 3. Antigen retrieval protocols were optimized with positive controls for each antibody. For co-immunofluorescent analysis, antigen retrieval corresponding to the respective αSyn antibody was performed as summarized in Table 3. For most antibodies, blocking was performed for 30 min with a 5% milk/0.1 M Tris, pH 7.6 and washing steps in 0.1 M Tris, pH 7.6. For antibody 1A2, blocking was performed with 5% milk/Tris buffered saline (TBS; 50 mM Tris, pH 7.5, 150 mM NaCl) followed by washing with TBS. Primary antibodies anti-CD11b (1:2000, Abcam) or rabbit anti-GFAP (1:2000, DAKO) were incubated with a respective anti-αSynΔC specific antibody (2G5, 1A2, 10A4, 5C1 or 2G7) and applied to slides for incubation overnight. Following overnight incubation, slides were washed 3 times for 10 min and then blocked for 30 min. Following blocking with 5% milk/0.1 M Tris, pH 7.6, secondary antibodies goat anti-mouse IgG Alexa Fluor 594 (1:500, Thermo Fisher) and goat anti-rabbit IgG Alexa Fluor 488 (1:500, Thermo Fisher) were diluted in 5% milk/0.1 M Tris, pH 7.6, applied to slides, and incubated for 2 h in the dark at room temperature. Slides were washed 2 times in a dark chamber for 20 min and then incubated in Autofluorescence Eliminator Reagent (Millipore) for 10 min. Excess Autofluorescence Eliminator Reagent was removed with 70% ethanol and slides were washed for 5 min in deionized water. Slides were then counterstained with 4′,6-diamidino-2-phenylindole (Invitrogen), washed once in deionized water for 5 min, and cover slipped with VECTASHIELD Antifade Mounting Medium (Vector Laboratories).

**Table 3:** Antibodies used for immunostaining and retrieval methods

Key resources table: immunohistochemistry/immunofluorescence					
Host	Identifier	Specificity	Antigen retrieval	Source	Reference
Rabbit	CD11b	Macrophages Activated Microglia		Abcam	
Rabbit	GFAP	Astrocytes		DAKO	
Mouse	3H19	αSyn (110–119)	DAKO Target Retrieval Solution, heat bath /70% formic acid	B. Giasson University of Florida College of Medicine; Florida; USA	[21]
Mouse	2G5	αSynΔC103	Formalin incubation/DAKO Target Retrieval Solution, heat bath /70% formic acid	B. Giasson University of Florida College of Medicine; Florida; USA	[14]
Mouse	1A2	αSynΔC114	DAKO Target Retrieval Solution, heat bath	B. Giasson University of Florida College of Medicine; Florida; USA	[33]
Mouse	10A4	αSynΔC122	DAKO Target Retrieval Solution, heat bath	B. Giasson University of Florida College of Medicine; Florida; USA	[14]
Mouse	5C1	αSynΔC125	DAKO Target Retrieval Solution, heat bath	B. Giasson University of Florida College of Medicine; Florida; USA	[14]
Mouse	2G7	αSynΔC129	DAKO Target Retrieval Solution, heat bath	B. Giasson University of Florida College of Medicine; Florida; USA	[14]

### Semi-quantification and digital analysis of pathology

All immunohistochemical stained sections were digitally scanned using an Aperio Scan Scope AT2 instrument (40 × magnification; Aperio Technologies Inc., Vista, CA, USA) and images of representative areas of pathology were captured using the ImageScope software (40 × magnification; Aperio Technologies Inc. Vista, CA, USA). Tissue sections were manually scored for αSyn pathology on a scale of 0 (no pathology) to 3 (highest pathology) by three independent raters using the Allen Brain Atlas to define regions (Allen Reference Atlas—Mouse Brain [brain atlas available from atlas.brain-map.org)]. One section per antibody, per animal were used for quantification. Scores were averaged and normalized [6, 13, 19, 27]. For immunofluorescent staining, representative images were captured at 40 × magnification using an Olympus BX51 fluorescence microscope mounted with a DP71 digital camera (Olympus). Colocalization of staining was assessed by two independent observers. Representative images were corrected for color/hue values; brightness/contrast adjustments were applied identically on captured images within each figure using Adobe Photoshop CS3 (Adobe Systems, San Jose, CA, USA). All raw files are available upon request. Representative color-gradient heat maps were generated using Microsoft excel, where normalized regional semiquantitative values (0–100) were associated with corresponding brain regions represented on an Allen Brain Atlas based template of outlined regions.

## Results

### Investigation of the regional distribution of the major forms of αSynΔC in the CNS of prion-type seeded αSyn transgenic mice

**Fig. 2:**
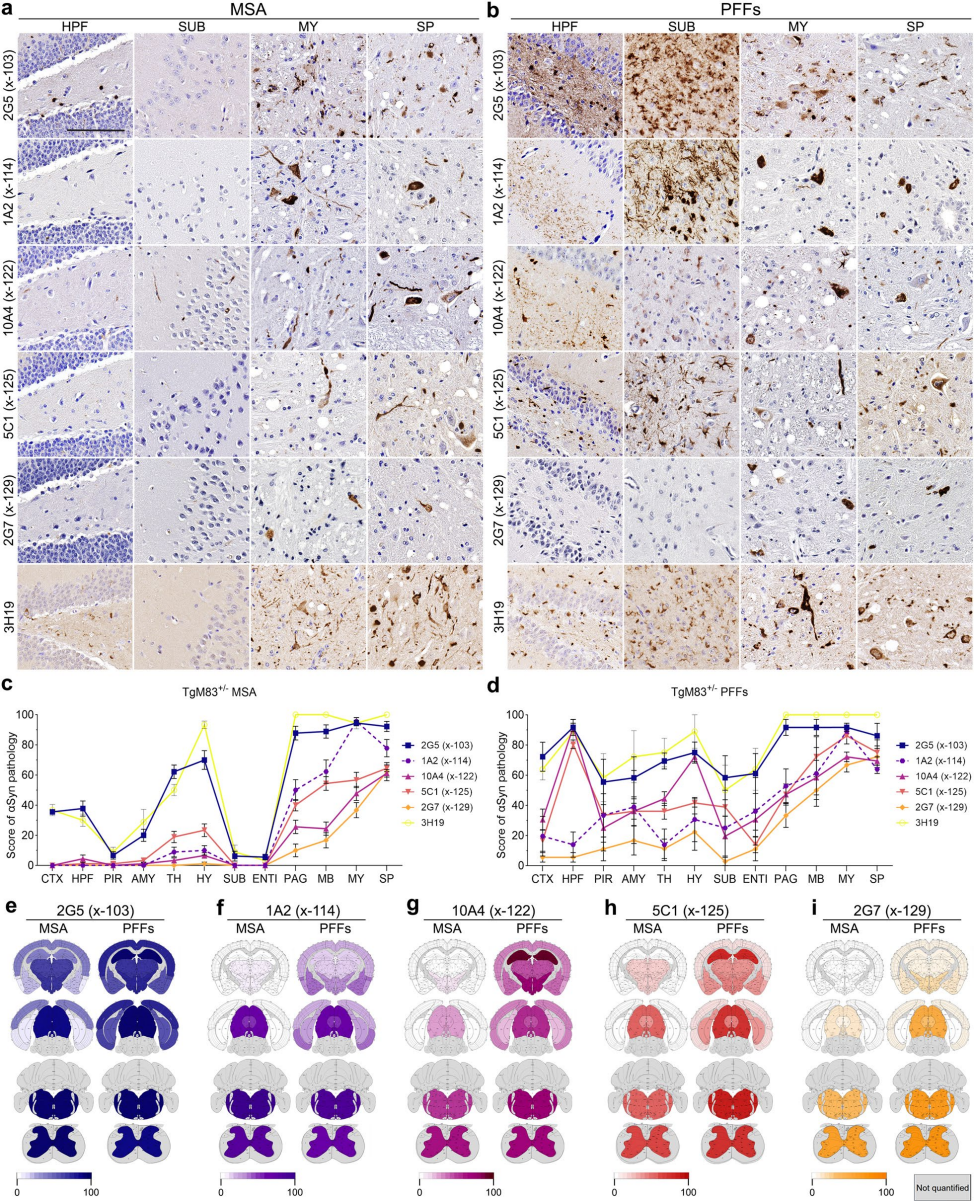
Comparison of strain specific regional deposition of αSynΔC in the CNS of TgM83^+/-^ mice. **a**, **b** Representative images of αSyn and αSynΔC immunohistochemical staining in TgM83^+/-^ mice seeded with (**a**) MSA lysates or (**b**) PFFs. Immunohistochemistry was performed with antibodies 3H19 (αSyn 110–119), 2G5 (αSynΔC103), 1A2 (αSynΔC114), 10A4 (αSynΔC122), 5C1 (αSynΔC125) or 2G7 (αSynΔC129). Selected brain regions, including HPF (hippocampal formation), SUB (subiculum), MY (medulla), and SP (spine) are depicted. Scale bar: 100 µm. **c**, **d** Semi-quantification comparing the regional distribution and burden of 3H19 and αSynΔC positive inclusions. Additional abbreviations: CTX (cortex), PIR (piriform cortex), AMY (amygdala), TH (thalamus), HY (hypothalamus), ENTI (entorhinal cortex), PAG (periaqueductal gray), and MB (midbrain). **e**–**i** Heatmap distributions of αSynΔC pathology

**Fig. 3:**
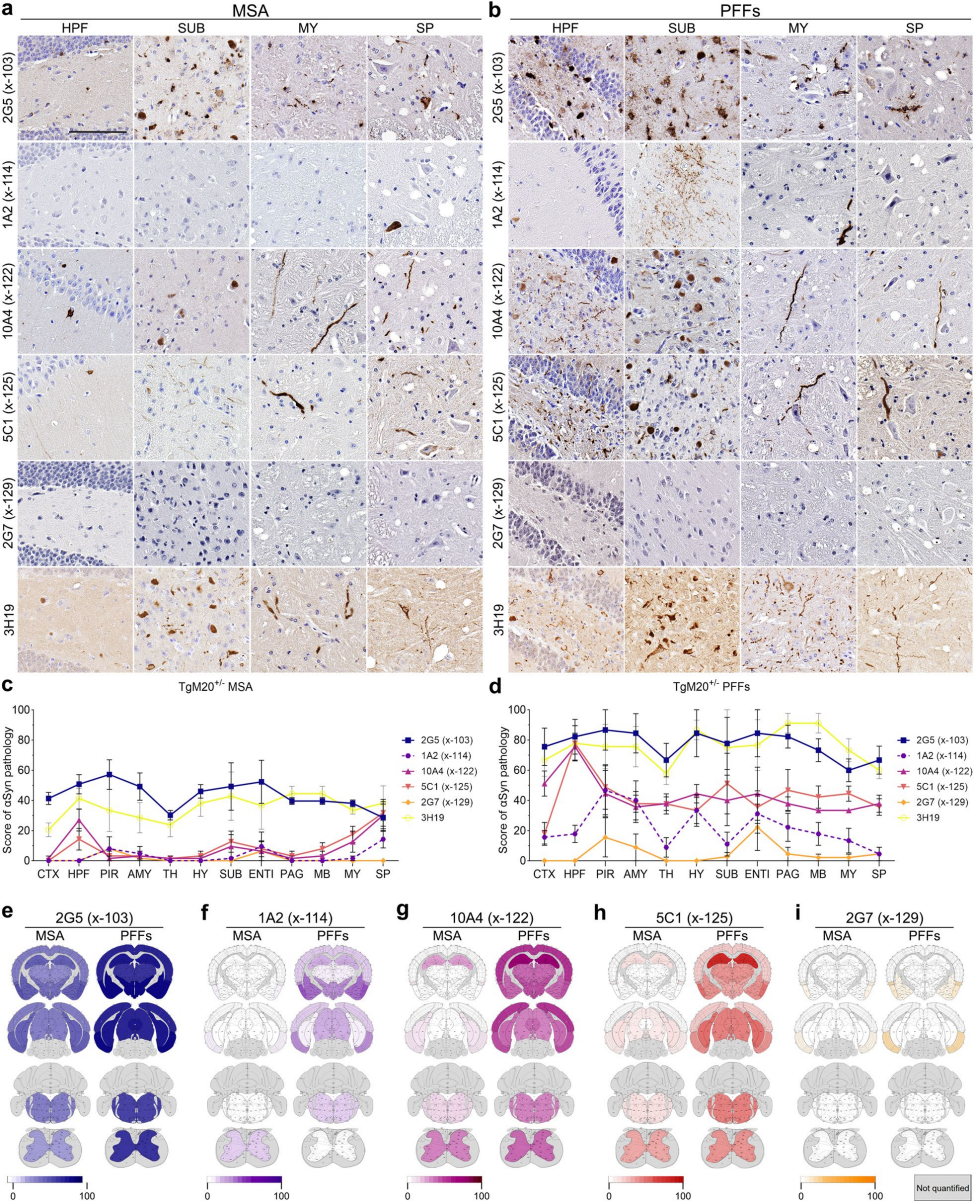
Comparison of strain specific regional deposition of αSynΔC in the CNS of TgM20^+/-^ mice. **a**, **b** Representative images of αSyn and αSynΔC immunohistochemical staining in TgM20^+/-^ mice seeded injected with (**a**) MSA lysates or (**b**) PFFs. Immunohistochemistry was performed with antibodies 3H19 (αSyn 110–119) 2G5 (αSynΔC103), 1A2 (αSynΔC114), 10A4 (αSynΔC122), 5C1 (αSynΔC125) or 2G7 (αSynΔC129). Selected brain regions, including HPF (hippocampal formation), SUB (subiculum), MY (medulla), and SP (spine) are depicted. Scale bar: 100 µm. **c**, **d** Semi-quantification comparing the regional distribution and burden of 3H19 and αSynΔC positive inclusions. Additional abbreviations: CTX (cortex), PIR (piriform cortex), AMY (amygdala), TH (thalamus), HY (hypothalamus), ENTI (entorhinal cortex), PAG (periaqueductal gray), and MB (midbrain). **e**–**i** Heatmap distributions of αSynΔC pathology

To investigate the comparative effects of MSA brain lysate and PFFs in inducing a range of αSynΔC pathology in TgM83^+/-^ and TgM20^+/-^ mice, we probed tissue from a prior report [21] using our panel of antibodies specific for αSyn C-terminally truncated at residues 103 (2G5), 114 (1A2), 122 (10A4), 125 (5C1) and 129 (2G7) (Fig. 1). We analyzed the regional distribution and burden of αSynΔC pathology in MSA and PFF injected TgM83^+/-^ and TgM20^+/-^ mice using a semiquantitative analysis and compared this to antibody 3H19, which is not truncation specific, as an indicator of global αSyn inclusion pathology (Figs. 2, 3). Immunohistochemical staining with antibody 3H19 and the αSynΔC specific antibodies in the CNS tissues from TgM83^+/-^ and TgM20^+/-^ mice injected with control human brain lysates did not reveal any inclusions (Supplemental Fig. 1)[21].

We found that both MSA lysate and PFF seeded TgM83^+/-^ animals exhibited inclusions positive for all αSynΔC variants (Fig. 2), with MSA injected TgM83^+/-^ mice displaying the greatest abundance of αSynΔC positive pathology in hindbrain regions, such as the medulla, and spine (Fig. 2a, c), and PFF-injected TgM83^+/-^ animals demonstrating robust levels of widespread, global pathology (Fig. 2b, d). Moreover, the spatial distribution of αSynΔC immunoreactive inclusions exhibited a correlation with the pattern observed for 3H19-positive αSyn pathology. This profile was closely followed in MSA-injected TgM83^+/-^ mice (Fig. 2c) although in PFF-injected TgM83^+/-^  mice this alignment was less pronounced (Fig. 2d). However, the comparative regional burden showed considerable variability. MSA-inoculated TgM83^+/-^ mice presented with lower levels of αSynΔC immunoreactive inclusions, except for 2G5, compared to 3H19 in corresponding regions. Interestingly, in the medulla, 2G5 and 1A2-positive inclusions were detected at a similar level to 3H19 (Fig. 2c). Throughout other regions, the relative levels of 2G5-positive pathology were consistent with those of 3H19. Apart from 2G5 staining, PFF-injected TgM83^+/-^ mice showed overall lower levels of αSynΔC immunoreactive inclusions compared to 3H19, in most corresponding regions. However, there were several regions wherein certain αSynΔC antibodies detected positive pathology at a similar level to 3H19, including 2G5, 10A4 and 5C1 in the hippocampus, 2G5 and 10A4 in the hypothalamus, 2G5 and 5C1 in the subiculum, and 2G5, 1A2 and 5C1 in the medulla (Fig. 2d). Comparison of the distribution profile of αSyn pathology with antibodies 3H19 and the antibodies specific for αSynΔC were similar when the mice injected with both MSA lysates were assessed separately (Supplemental Fig. 2).For a detailed regional analysis of αSynΔC pathology between inoculum, heatmaps were generated, corresponding to each αSynΔC truncation antibody measured in this study (Fig. 2e–i). In PFF-injected TgM83^+/-^ animals overall 2G5 positivity surpassed that in MSA-injected counterparts, reaching the highest burden in the hippocampus and comparable levels in the midbrain, medulla, and spine (Fig. 2e). 1A2 positivity was moderate to high in both MSA-injected and PFF-injected TgM83^+/-^ animals in the midbrain, medulla, and spine; notably, only PFF-injected mice exhibited 1A2 positivity in cortex and amygdalar regions (Fig. 2f). 10A4 positivity was detected in hippocampus, amygdala, and cortex of PFF-injected TgM83^+/-^ mice only, but medullar and spinal 10A4 positive pathology was observed at similar levels in both cohorts (Fig. 2g). 5C1 positive pathology exhibited a similar trend to that of 10A4, with high to moderate levels of pathology detected in the hippocampal, amygdalar and cortical regions in PFF-injected TgM83^+/-^ mice only and similar, albeit lower in MSA-injected mice, in the midbrain, medulla and spine (Fig. 2h). Finally, heatmaps of 2G7 pathology revealed overall higher levels of 2G7 pathology in PFF-injected TgM83^+/-^ mice, with the burden of pathology becoming more similar between cohorts in the hindbrain and spinal regions (Fig. 2i).

In the TgM20^+/-^ seeded mouse models, the distribution and abundance of 2G5 were notably more robust compared to other αSyn C-terminal truncation-specific antibodies, approaching the signal of the non-truncation-specific antibody 3H19 (Fig. 3). Animals inoculated with MSA lysates exhibited inclusions positive for antibodies 2G5, 10A4, and 5C1 in the hippocampus, subiculum, medulla, and spine, with predominantly spine-associated 1A2-positive inclusions, while 2G7 was virtually undetected in all regions (Fig. 3a, c). In contrast, TgM20^+/-^ animals injected with PFFs exhibited inclusions positive for antibodies 2G5, 1A2, 10A4 and 5C1 in the hippocampus, subiculum, and medulla (Fig. 3b, d).

We next conducted an in-depth analysis of αSynΔC pathology distribution and burden in TgM20^+/-^ mice injected with MSA lysate and PFFs. In both M20 cohorts, 2G5-positive pathology closely mirrored 3H19 pathology, evident across all assessed CNS regions. Notably, MSA lysate-injected TgM20^+/-^ mice exhibited lower pathology levels compared to PFFs (Fig. 3c, d). In most CNS regions quantified for MSA lysate-injected TgM20^+/-^ mice, pathology indicated by other αSynΔC-specific antibodies was notably lower, except for the spine where 10A4 and 5C1 positive inclusions were abundant (Fig. 3c). Additionally, PFF seeding in TgM20^+/-^ mice resulted in a more widespread presentation of most forms of αSynΔC, with 2G7 being relatively low across all regions except for the entorhinal cortex (Fig. 3d).

To further distinguish the regional differences in αSynΔC pathology between inoculum, heatmaps were generated, corresponding to each αSynΔC truncation antibody measured in this study (Fig. 3e–i). 2G5 positivity was overall higher in PFF-injected TgM20^+/-^ animals compared to MSA-injected TgM20^+/-^ animals (Fig. 3e). Although 1A2 positivity was not as robust as other αSynΔC, it revealed a clear difference between MSA and PFFs seeding. In MSA lysate injected mice, 1A2 immunoreactivity was predominantly observed in the spine, while in PFF-injected TgM20^+/-^ animals 1A2 positivity was more widespread and moderate in the piriform/entorhinal cortex, amygdala, and hypothalamus, with little or no 1A2 pathology in the spine (Fig. 3f). In PFF-injected TgM20^+/-^ mice, 10A4 positivity also demonstrated higher burden of pathology compared to MSA lysate seeded mice but the spine was comparable between cohorts (Fig. 3g). 5C1 reactive pathology exhibited a similar trend to that of 10A4, with high to moderate levels of pathology detected in the hippocampal, amygdalar, cortical, midbrain and medullar regions in PFF-injected TgM20^+/-^ mice only and comparable 5C1 positive burden in the spine of MSA lysate and PFF-injected mice (Fig. 3h). 2G7 pathology was low in both MSA lysate and PFF-injected TgM20^+/-^ mice with similar distribution but overall higher levels of 2G7 pathology in PFF-injected TgM20^+/-^ mice, with the burden of pathology restricted to the forebrain (Fig. 3i).

### Assessment of the spectrum of αSynΔC found within astrocytes of αSyn transgenic mouse seeded with MSA lysates or PFFs

**Fig. 4:**
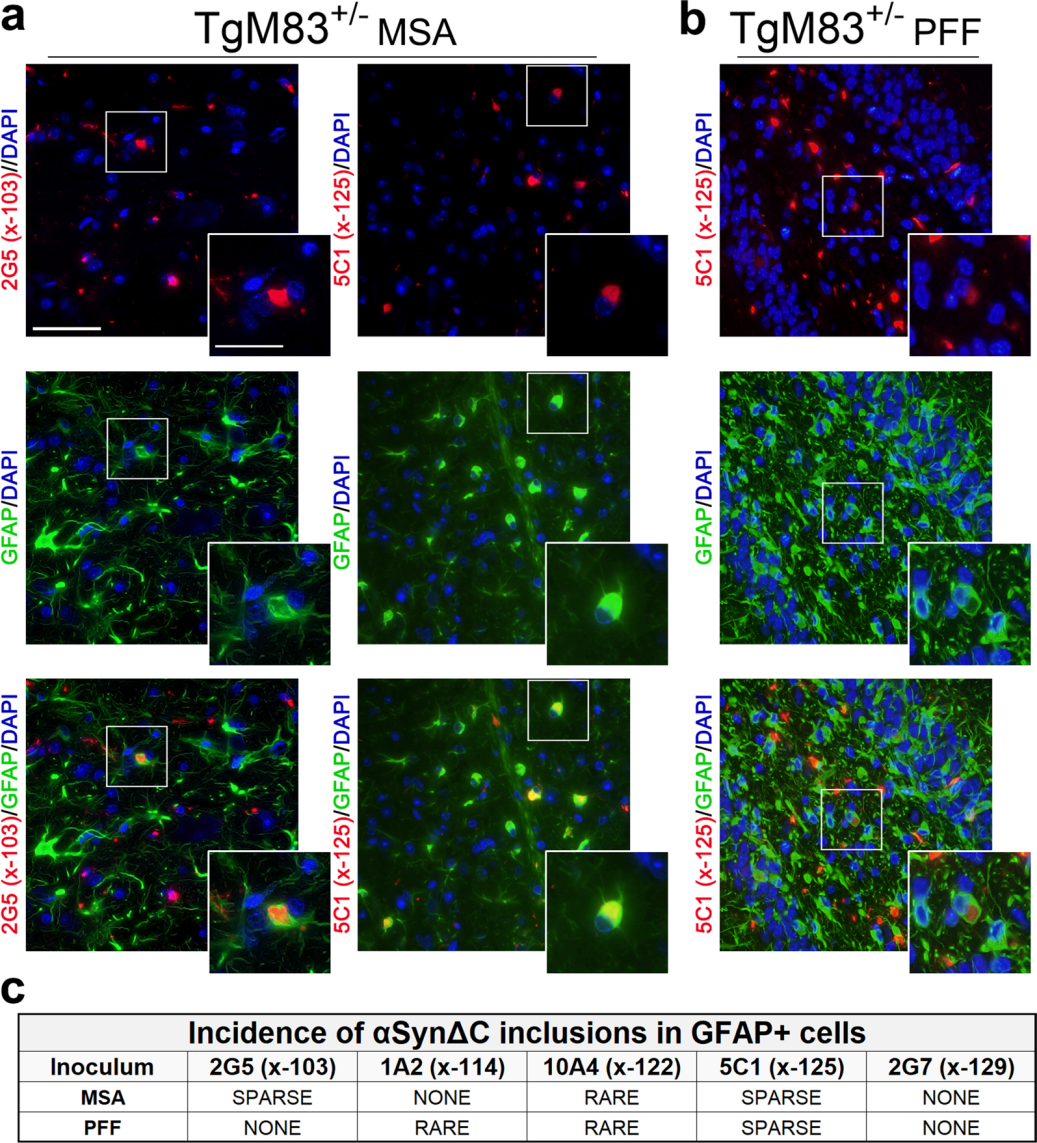
Astrocytic αSynΔC inclusion pathology in TgM83^+/-^ mice seeded with MSA lysates and PFFs. Representative co-immunofluorescence images from TgM83^+/-^ mice brain sections seeded with (**a**) MSA lysates or (**b**) PFFs. Sections were double-labeled with GFAP, an astrocyte marker, and αSynΔC-specific antibodies 2G5 (αSynΔC103), 1A2 (αSynΔC114), 10A4 (αSynΔC122), 5C1 (αSynΔC125) or 2G7 (αSynΔC129). Scale bars: 50 µm; 25 µm (insets). **c** Images were assessed based on the frequency of αSynΔC variants co-localizing with GFAP+ cells, categorized as frequent, sparse, rare, or absent (none). The images shown highlight αSynΔC variants that were observed sparsely within GFAP+ cells. Representative images depicted are of the medulla (2G5 in **a**), the periaqueductal grey areas (5C1 in **a**), and the hippocampal formation (5C1 in **b**)

Our previous investigation of prion-type seeding in αSyn transgenic mice revealed that MSA injection in TgM20^+/-^ mice can result in pSer129-positive αSyn inclusions within astrocytes, however, this phenomenon was not detected in TgM83^+/-^ mice [21]. We therefore investigated whether αSynΔC positive inclusions would be detected in astrocytes in TgM83^+/-^ and TgM20^+/-^ mice after MSA or PFF inoculation. Sections were co-stained using an antibody for glial fibrillary acidic protein (GFAP), an astrocytic protein whose expression is upregulated upon activation [48] and our panel of αSynΔC-specific antibodies. Astrocytes in MSA lysate-injected TgM83^+/-^ mice sparsely colocalized with 2G5 and 5C1 positive inclusions (Fig. 4), whereas astrocytes in PFF-injected TgM83^+/-^ mice occasionally exhibited 5C1 positive inclusions but were not found to have 2G5 reactive inclusions (Fig. 4). Astrocytes with these αSynΔC positive inclusions appeared distended, the inclusions were in the cell soma, while the nuclei appeared normal (Fig. 4a, b). Interestingly, astrocytes with 5C1 positive inclusions appear to exhibit reduced projections and, in some cases, displaced GFAP staining (Fig. 4a, b; insets). Both cohorts exhibited rare 10A4 positive astrocytic inclusions, and PFF-injected, but not MSA-injected, TgM83^+/-^ mice exhibited rare 1A2 positive astrocytic inclusions (Fig. 4c).

**Fig. 5:**
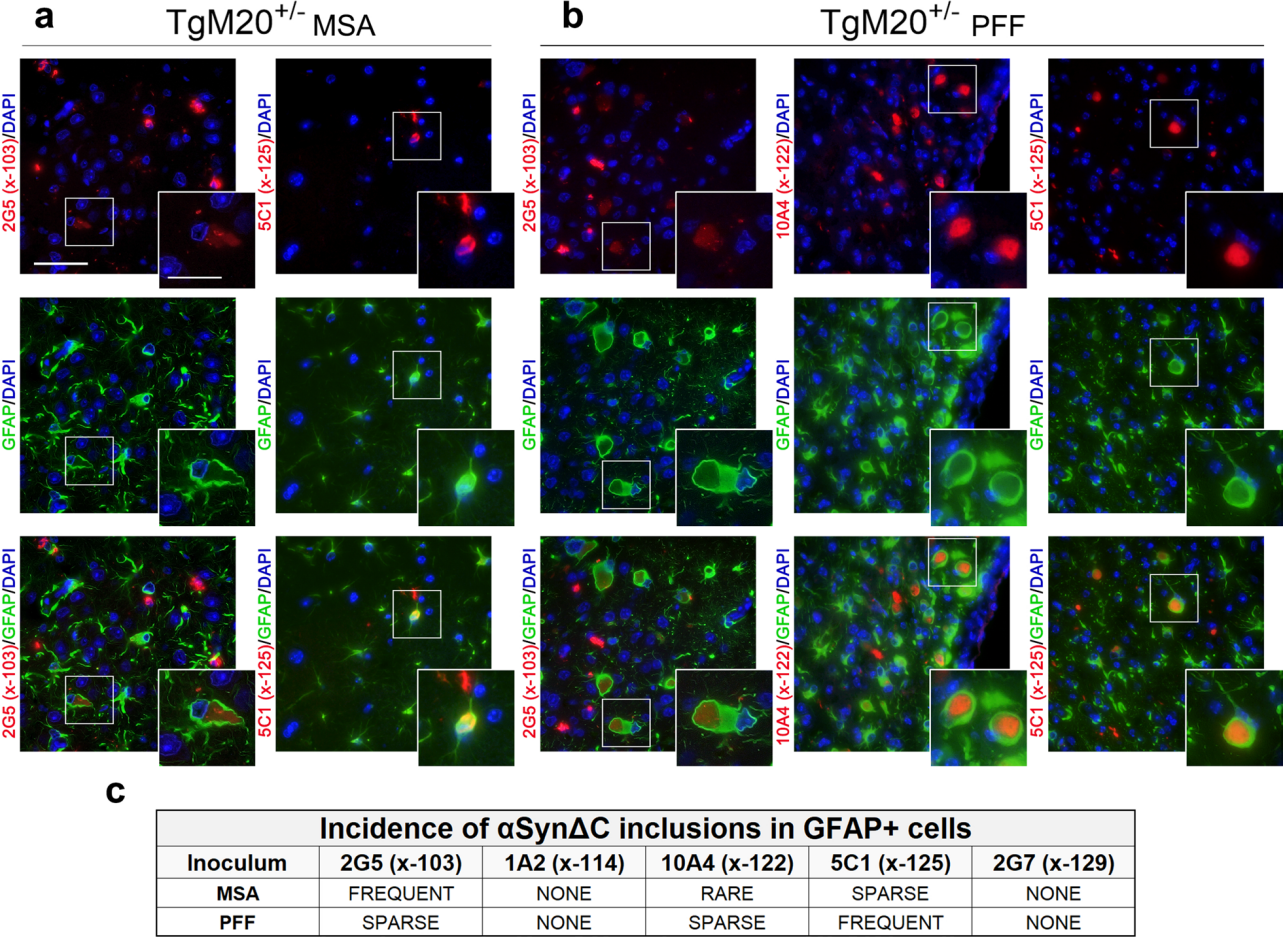
Astrocytic αSynΔC Positive Pathology in TgM20^+/-^ Mice Seeded with MSA Lysate and PFFs. Representative co-immunofluorescence images from TgM20^+/-^ mice brain sections seeded with (**a**) MSA or (**b**) PFFs. Sections were double-labeled with GFAP, an astrocyte marker, and αSynΔC-specific antibodies 2G5 (αSynΔC103), 1A2 (αSynΔC114), 10A4 (αSynΔC122), 5C1 (αSynΔC125), 2G7 (αSynΔC129). Scale bars: 50 µm; 25 µm (insets). **c** Images were assessed based on the frequency of αSynΔC variants co-localizing with GFAP+ cells, categorized as frequent, sparse, rare, or absent (none). The images shown highlights αSynΔC variants that were observed either frequently or sparsely within GFAP+ cells. Representative images depicted are of the subiculum (2G5 in **a** and 2G5 in **b**), the hippocampal formation (5C1 in **a**), and the entorhinal cortex (10A4 in **b** and 5C1 in **b**)

In TgM20^+/-^ seeded mice, GFAP reactive astrocytes exhibited a higher burden and greater diversity in αSynΔC species compared to TgM83^+/-^ mice. TgM20^+/-^ mice injected with MSA lysate resulted in frequent 2G5 positive astrocytic inclusions, sparse 5C1 astrocytic inclusions and rare 10A4 astrocytic inclusions (Fig. 5). PFF induced pathology in TgM20^+/-^ mice resulted in astrocytic inclusions with sparse 2G5 and 10A4 positivity and frequent 5C1 positivity (Fig. 5). Notably, astrocytes containing 2G5 positive inclusions appeared aberrant in morphology, typically exhibiting oblong, distorted cell bodies, reduced processes and displaced GFAP staining (Fig. 5a, b; insets). GFAP stained astrocytes with 10A4 or 5C1 positive inclusions appeared to have reduced branching but retained round, normal nuclei (Fig. 5a, b; insets). In PFF-injected TgM20^+/-^ mice, astrocytes containing 10A4 and 5C1 positive inclusions appeared more globose with displaced GFAP staining (Fig. 5b; insets). In summary, in all mice cohorts, astrocytic inclusions showed immunoreactivity for 10A4 and 5C1, and all cohorts exhibited 2G5-immunoreactive astrocytic inclusions, except PFF-injected TgM83^+/-^ mice which was the only cohort positive for 1A2-immunoreactive astrocytic inclusions.

### Abundant αSynΔC-103 positive inclusion pathology in CD11b reactive CNS cells of prion-type seeded αSyn transgenic mice

Previous in vivo studies, employing transgenic overexpression and viral vector-based seeding models, consistently reported the presence of αSyn in activated microglia [5, 36, 38, 45]. However, despite microglia exhibiting a strong potential for phagocytic activity and αSynΔC being a common PTM in human diseased brains, few reports have detailed the nature of microglial αSynΔC inclusions [45]. Therefore, we wanted to investigate the prevalence and characteristics of αSynΔC within microglia, using our panel of antibodies specifically designed for a spectrum of αSynΔC and using CD11b as a marker of activated microglia in the CNS parenchyma.

**Fig. 6:**
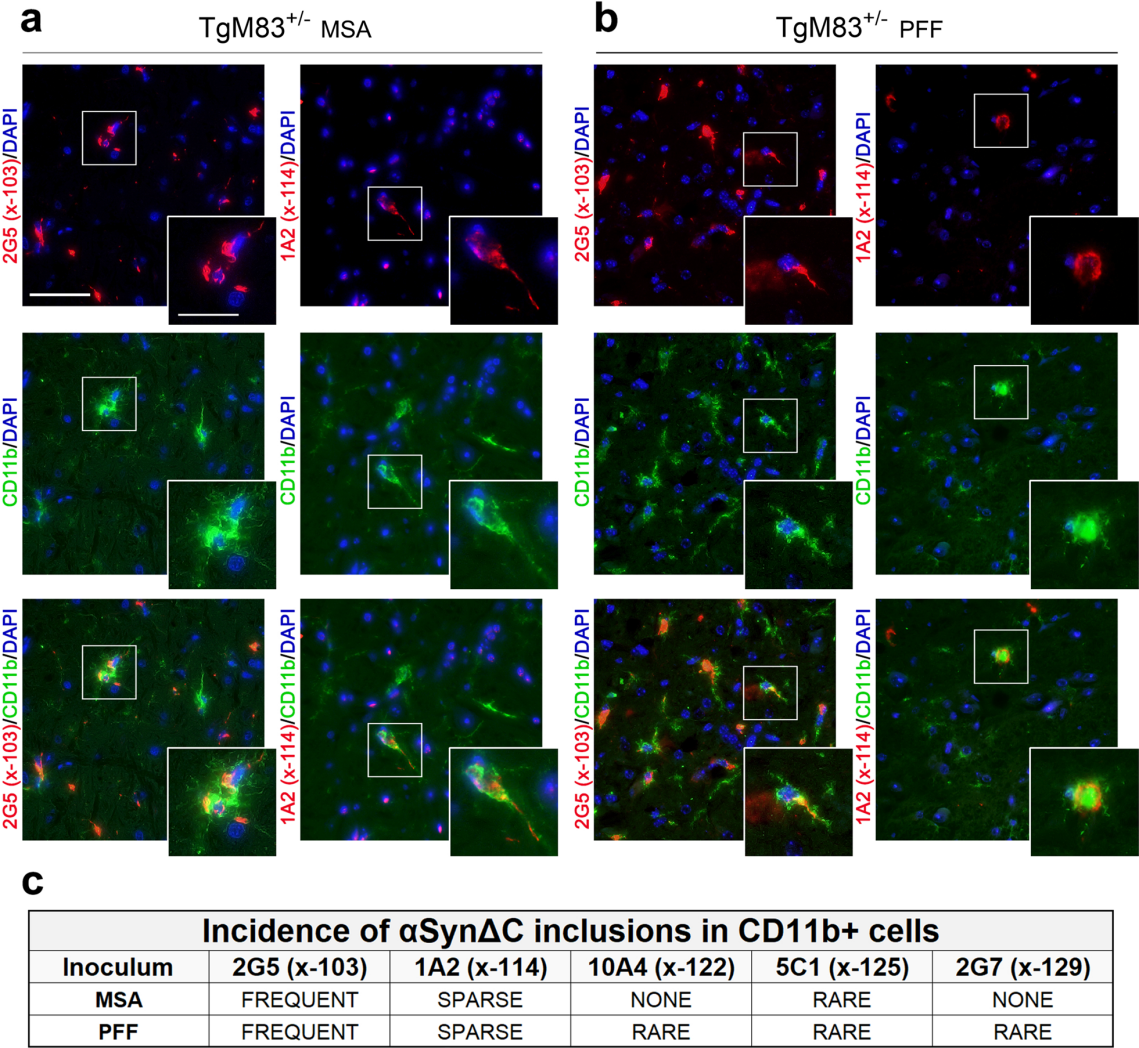
αSynΔC Inclusion Pathology within CD11b Reactive Cells in the CNS of TgM83^+/-^ Mice Seeded with MSA Lysates and PFFs. Representative co-immunofluorescence images from TgM83^+/-^ mice brain sections seeded with (**a**) MSA or (**b**) PFFs. Sections were double-labeled with CD11b, a marker of activated microglia, and αSynΔC-specific antibodies 2G5, (αSynΔC103), 1A2 (αSynΔC114), 10A4 (αSynΔC122), 5C1 (αSynΔC125) and 2G7 (αSynΔC129). Scale bars: 50 µm; 25 µm (insets). **c** Images were assessed based on the frequency of αSynΔC variants co-localizing with CD11b+ cells, categorized as frequent, sparse, rare, or absent (none). This figure highlights αSynΔC variants that were observed either frequently or sparsely within CD11b+ cells. Representative images depicted are of the midbrain (2G5 in **a**) and the medulla (1A2 in **a** and **b** and 2G5 in **b**)

In both MSA-injected and PFF-injected TgM83^+/-^ mice, we observed a notable presence of microglia positive for 2G5 and 1A2 αSyn inclusions, with varying frequencies: frequent in the former and sparse in the latter (Fig. 6). MSA-injected TgM83^+/-^ mice displayed rare occurrences of 5C1 pathology in microglia, whereas PFF-injected TgM83^+/-^ mice displayed rare occurrences of 10A4, 5C1, and 2G7 pathology in microglia (Fig. 6). Microglia that colocalized with 2G5 ranged in morphology, with most appearing to have hypertrophic cell bodies and shortened appendages (Fig. 6a) that were sometimes prickly in appearance (Fig. 6b). Other morphologies include an ameboid or globular shape with condensed or nonexistent processes. The 2G5 positive microglia in these animals were frequently overlapping, varied heavily in spatial patterning, often forming nodules with multiple nuclei in proximity (Fig. 6a). 2G5 positivity was intermittently detected in rod-like microglia, which exhibited thin, elongated somas and reedy, barbed processes, wherein which the 2G5 positivity was detected. Comparatively, 1A2 positive microglia exhibited a more restricted range of morphologies, typically appearing with hypertrophic cell bodies and withdrawn processes (Fig. 6a, b).

**Fig. 7:**
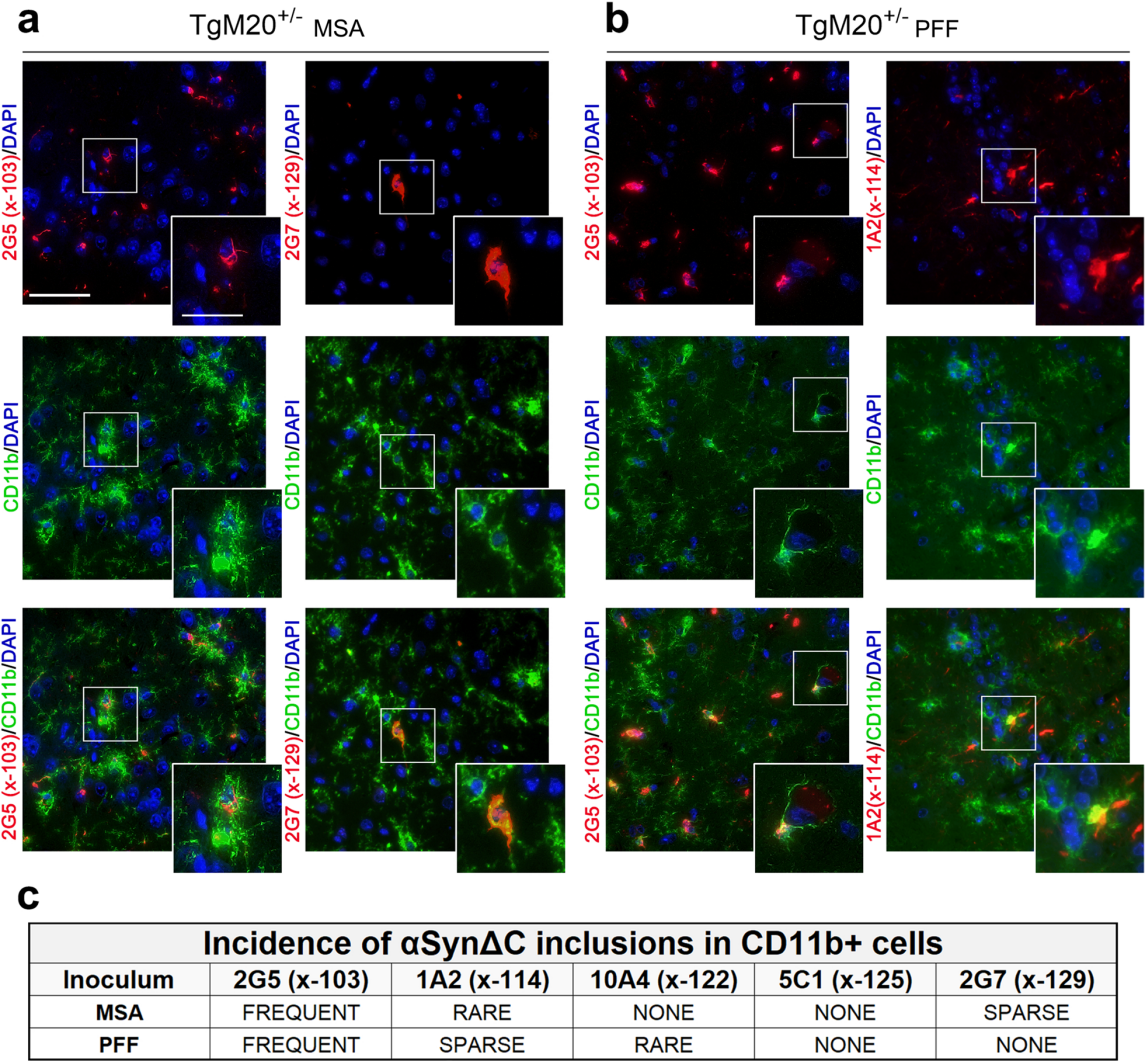
αSynΔC inclusion pathology within CD11b reactive cells in the CNS of TgM20^+/-^ mice seeded with MSA lysates and PFFs. Representative co-immunofluorescence images from TgM20^+/-^ mice brain sections seeded with (**a**) MSA or (**b**) PFFs. Sections were double-labeled with CD11b, a marker of activated microglia, and αSynΔC-specific antibodies 2G5 (αSynΔC103), 1A2 (αSynΔC114), 10A4 (αSynΔC122), 5C1 (αSynΔC125) and 2G7 (αSynΔC129). Scale bars: 50 µm; 25 µm (insets). **c** Images were assessed based on the frequency of αSynΔC variants co-localizing with CD11b+ cells, categorized as frequent, sparse, rare, or absent (none). This figure highlights αSynΔC variants that were observed either frequently or sparsely within CD11b+ cells. Representative images depicted are of the piriform cortex (2G5 in **a** and 1A2 in **b**), the entorhinal cortex (2G7 in **a**), and the subiculum (2G5 in **b**)

In seeded TgM20^+/-^ mice, double labeling with CD11b and our panel of αSynΔC-specific antibodies unveiled an abundance of 2G5-positive inclusions in both MSA lysate- and PFF-injected TgM20^+/-^ mice (Fig. 7). Conversely, a sparser presentation of 1A2-positive inclusions was observed in TgM20^+/-^ mice treated with PFFs (Fig. 7). Remarkably, in both TgM20^+/-^ cohorts, 2G5 positive microglia infrequently exhibited the hypertrophic cell bodies that were characteristic in TgM83^+/-^ mice. In fact, in MSA-injected TgM20^+/-^ mice, microglia often appeared homeostatic, featuring sprawling, richly reticulated processes, despite harboring perinuclear threads of 2G5-positive inclusions (Fig. 7a).

In PFF-injected TgM20^+/-^ mice, 2G5 positive CD11b+ cells also maintained richly brachiated, albeit thicker and thornier, processes, accompanied by the presence of perinuclear inclusions (Fig. 7b). Remarkably, CD11b+ /2G5+ cells in this cohort were frequently observed in close contact with CD11b-/2G5+ cells. The processes of the CD11b+  cells often appear in contact with the soma of adjacent CD11b-/2G5+ cells, as shown in Fig. 7b (inset). Of note, the 2G5+ inclusions in the swollen soma of the non-CD11b+ cell exhibit a markedly different morphology compared to those found in the CD11b+ cell (Fig. 7b). In MSA-injected TgM20^+/-^ mice (Fig. 7a, c), 2G7-positive inclusions were sparsely observed within CD11b+ cells.

## Discussion

Multiple studies have provided compelling evidence supporting the presence of distinct αSyn strains, reminiscent of the characteristics observed in prions [4, 10, 28–30, 32, 35, 40, 47]. The predominantly unstructured nature of αSyn and its highly flexible, acidic C-terminus region is compatible with a multitude of molecular substructures leading to a spectrum of fibrillization kinetics that could be associated with diverse αSyn cellular toxicity properties. Indeed, variations in the structural features of the C-terminal region among αSyn strains lead to diverse effects on proteasome activity [44]. Proteolytic processing of αSyn at the C-terminus poses various potential routes towards cellular toxicity, for example, the partial or total removal of this acidic region heightens the likelihood of aggregation by increasing the net hydrophobicity of the protein (reviewed in [41]). Furthermore, increased levels of αSynΔC have been identified in the brain tissue of individuals with PD [24]. Our lab has demonstrated, using our recently developed panel of αSynΔC-specific antibodies targeting residues 103, 114, 122, 125, and 129, not only the prevalence of αSynΔC in post-mortem tissues of MSA, DLB and AD/ALB [14, 33] but also revealed its presence in the early stages of αSyn pathogenesis in TgM83^+/-^ mice seeded intramuscularly with PFFs [20].

We therefore wanted to continue this analysis of αSynΔC pathology in experimental models, to investigate factors that alter αSynΔC pathological profiles. We previously observed that the intrahippocampal administration of MSA-derived brain lysate or PFFs in TgM83^+/-^ and TgM20^+/-^ mice revealed extensive but regionally distinct patterns of pSer129-positive αSyn pathology. The ultimate severity of disease progression was found to be dictated by endogenously expressed αSyn, with severe motor impairment manifesting exclusively in TgM83^+/-^ mice [21]. In this study, we investigated potential strain-specific proteolytic patterns between TgM83^+/-^ and TgM20^+/-^ mice seeded with MSA-derived brain lysate or PFFs. We found that in TgM83^+/-^ prion-type seeded murine models, the regional pattern of C-terminally truncated αSyn generally corresponds with the prominence of 3H19-positive inclusions in the neuroaxis. PFF seeds resulted in more widespread inclusion pathology compared to MSA derived seeds in TgM83^+/-^ mice. For example, the entorhinal cortex and subiculum regions had modest pathology in MSA seeded mice. αSyn cleaved at residue 103 was the most robust and widespread αSynΔC modification observed and it tracked closely with overall αSyn inclusion pathology. Some brain regions presented with fewer types of αSyn cleavage products. For example, the brain stem regions presented with more abundant and diverse assortment of αSynΔC specific compared to the cortex. Our studies did not investigate the temporal presentation of αSynΔC products relative to inclusion formation, so it remains unclear if these differences are due to region selective vulnerability to generate and accumulate specific forms of αSynΔC or temporal progressive outcomes associated with the initiation and spread of αSyn pathology.

Our studies in TgM20^+/-^ mice also revealed greater levels of αSynΔC burden across all investigated regions following PFF injection; this contrasted with the diminutive αSynΔC-positive burden detected in their MSA-injected counterparts, underscoring distinct neuropathological responses by these different prion-type seeds. Furthermore, the profiles of induced αSyn pathology and αSynΔC in the neuroaxis of MSA induced TgM20^+/-^ and TgM83^+/-^ mice were distinct. As the latter mice express human αSyn with the A53T mutation differentiating TgM83^+/-^ mice from TgM20^+/-^ mice, these data imply that this relatively subtle differences in the N-terminal region of αSyn can impact the presentation of the C-terminal region to protease within inclusions, which could reflect differences in the molecular folding of αSyn within amyloid fibrils. Alternatively, αSynΔC species could be generated during the process of prion-type cellular transmission with WT compared to A53T αSyn generating altered proteolytic profiles and these differences could influence the temporal and regional spread of αSyn pathology in addition to preferential accumulation of different types of αSynΔC. It should be noted that the age at termination differs between TgM83^+/-^ and TgM20^+/-^ mice, both in terms of time since birth and months post inoculation. It may be the case that these factors may have contributed to the differences in the pattern and burden of αSynΔC observed in TgM20^+/-^ mice compared to TgM83^+/-^ mice. Nevertheless, these findings suggest a strain-specific differential proteolytic processing of αSyn aggregates, with the MSA strain yielding a more tightly controlled efflux of αSynΔC variants in abundance of distinct variants, extent of regions affected and overall burden of variant pathology. In contrast the PFF strain produces a much more robust array of αSynΔC conformers, affects a greater number of brain regions and results in, on average, a higher burden of pathology.

A limitation in our analysis of regionally distributed αSynΔC conformers in these cohorts is the inherent temporal constraint. The presented data provides a static snapshot, offering limited insight into the dynamic timeline of αSynΔC variant production. This limitation could be addressed through future studies incorporating designated endpoints aligned with distinct stages of disease progression. For instance, the identification of the primary site of significant αSynΔC variant generation remains enigmatic. While it is reasonable to hypothesize that initiation occurred at the injection site, further exploration is warranted, particularly in understanding the fate of generated αSyn truncations in cohorts lacking hippocampal αSynΔC-positive pathology. This highlights a potential host/inoculum-dependent aspect of αSynΔC proteolytic processing that remains unresolved in our study. Future investigations should strategically include early timepoints post-injection, providing an adequate timeframe for the endocytosis of exogenous αSyn fibrils by surrounding cells, without allowing for extensive intercellular αSyn pathology development.

In our prior study, an intriguing role of astrocytes in prion-type seeding of αSyn transgenic mice emerged, with MSA injection leading to pSer129-positive αSyn astrocytic inclusions in TgM20^+/-^ mice but not TgM83^+/-^ mice, while PFF inoculation led to pSer129-positive αSyn astrocytic inclusions in both cohorts [21]. Therefore, in the current study, we also explored the occurrence of αSynΔC-positive inclusions in the astrocytes of the MSA and PFFs seeded cohorts of mice. These studies revealed variations in astrocytic αSynΔC type but significant host and inoculum-dependent differences in astrocytic αSynΔC burden. In all examined cohorts, astrocytic inclusions consistently displayed immunoreactivity for 10A4 and 5C1. Almost all cohorts exhibited 2G5-immunoreactive astrocytic inclusions, except for PFF-injected TgM83^+/-^ mice. Notably, this cohort was unique in having positive, albeit rare, 1A2-immunoreactive astrocytic inclusions, highlighting distinct immunoreactive profiles associated with different seeding and transgenic conditions.

In addition, the exploration of αSynΔC-positive inclusions in microglia revealed intriguing patterns of pathology across cohorts. In TgM83^+/-^ mice, MSA inoculation resulted in microglia with 2G5, 1A2 and 5C1 immunoreactivity, whereas in PFF-injected mice, we observed every variant of αSynΔC in microglia. Conversely, in TgM20^+/-^ mice, both seeding types resulted in microglia with 2G5 and 1A2 immunoreactive inclusions. However, MSA-inoculated animals also showed microglia with 2G7-positive inclusions, whereas PFF-inoculated mice displayed microglia with 10A4-positive inclusions. Despite substantial variation in the burden of different αSynΔC-positive inclusions in microglia, the consistent presence of certain αSynΔC variants across cohorts suggests a preference for 2G5 and 1A2 positive inclusions in microglia. Additionally, an inoculum-dependent variation was observed, with PFF injection resulting in greater diversity in proteolytically processed αSyn within CD11b+ cells. It should be noted that our study of glial cell involvement is limited by the markers used. GFAP is not a comprehensive astrocytic marker and therefore may not reveal astrocytes that are not GFAP+ . Similarly, CD11b only stains a subset of microglia.

It is possible that glial cells assume a crucial, though not fully elucidated, role in shaping the observed heterogeneity and spread in synucleinopathies. Notably, N- and C-terminally truncated astrocytic αSyn have need reported in the limbic cortical regions of DLB [1] and antibodies to the middle region of αSyn provide the ability for extensive detection of abundant astrocytic αSyn pathology in DLB [42]. Furthermore, microglia can be actively engaged in phagocytosing and degrading αSyn through autophagy [5, 38], directly providing a potential molecular pathway for strain influence. Cell type specific proteolytic processing has the potential to modify the biophysical properties of aggregated αSyn, setting the stage to alter quaternary structures directly or indirectly (via further conformational amplification). Spectral analysis using the fluorescent dye pentamer formyl thiopene acetic acid (pFTAA) has highlighted conformational distinctions between neuronal and microglial inclusions in four distinct transgenic mouse models of synucleinopathies. This distinction is speculated to arise from the removal of the C-terminus following microglial phagocytosis of neuron-released αSyn [45].

An important aspect of using αSynΔC specific antibodies as tools for pathological assessment is that the routine staining for pSer129 αSyn cannot detect these modified forms of αSyn. A striking commonality evident across all cohorts is the notable burden of αSynΔC103 observed throughout the neuroaxis and its cell-type promiscuity. Among αSynΔC variants, 2G5 immunoreactivity stood out as the most abundantly observed and was frequently observed in CD11b+ microglial cells. These surprising findings suggest that proteolytic processing of αSyn at residue 103 may occur at a rate that is dictated more by overall burden of aggregated αSyn inclusions and be a feature that is mostly host and inoculum independent. This may be the case if proteolytic processing of αSyn at residue 103 occurs predominantly after αSyn aggregation and fibril formation.

Alternatively, the αSynΔC103 variant could potentially signify a discrete aggregating form of αSyn that cannot be further processed. This may result in a particular strain with a domain role in determining the trajectory of αSyn inclusion formation through the initiation of nucleation events. Indeed, fibrils containing αSynΔC103 have demonstrated heightened templating ability, showcasing the capacity to propagate their unique, highly twisted conformational structure onto αSyn, thus forming heterotypic fibrils [17, 26, 41].

It can be hypothesized that the formation of this potent αSynΔC103 strain may be initiated in microglia, which are recognized for their superior efficiency in the intracellular uptake and degradation of exogenous αSyn in vitro [18]. It is possible that microglia endocytose most of the inoculum at the injection site resulting in the incomplete digestion and the formation of various αSynΔC, with αSynΔC103 being a dominant form.

The process of αSynΔC103 formation might be influenced by factors unique to the intracellular milieu, such as level of expression of enzymes targeting αSyn at residue 103, processivity of these enzymes, as well as abundance of substrates competing for the active site of said enzymes. ﻿For example, asparagine endopeptidase (AEP), a lysosomal cysteine cathepsin that cleaves αSyn at asparagine 103 and is found to be elevated in PD brains [49] has a speculative proclivity for partially degrading αSyn into αSynΔC conformers [41]. Intriguingly, AEP exhibits increased activity under conditions of oxidative stress [49], positioning it as an interesting and relevant target for experiments aimed at altering the production rate of αSynΔC conformers and assessing the impact on disease progression. Hypothetically, the generation of αSynΔC103 may act as a precipitating event, instigating a more virulent strain and expediting the rate of αSyn pathogenesis. In sharp contrast, the primary generation of another αSynΔC variant, such as αSynΔC129, may result in a slower manifestation of αSyn pathogenesis. This divergence may stem from potential incompatibility between αSynΔC129 fibrils and the surrounding full-length αSyn monomers. Crucially, the biophysical attributes of αSynΔC103, encompassing its small size, lack of C-terminus, diverse morphology, and widespread distribution across various regions and cell types within the cohorts investigated in this study, collectively imply its potential role as a 'super seed'—a versatile and ubiquitous factor exerting a profound influence on the dynamics of αSyn pathology. Moreover, the emergence of αSynΔC103 as a strain incubated within microglia could potentially provide an alternative interpretation for the transsynaptic spread of αSyn pathology. Existing evidence strongly supports neuroanatomically connected pathways as a major, albeit not exclusive, route for the propagation of αSyn inclusion pathology [15, 20, 23, 34, 46]. This alternative hypothesis posits the propagation of αSyn pathology through myeloid cells, potentially circumventing the conventional trans-synaptic pathway. In summary, our observations highlight the significant burden, extensive regional distribution, and cell-type promiscuity of αSynΔC103-positive inclusions as a compelling aspect of αSynΔC pathology. Additionally, we have proposed potential mechanisms that could explain our findings. Future investigations are imperative to elucidate whether αSynΔC103 and other αSynΔC variants indeed acts as potent initiating factors that nucleate pathogenesis and whether the hypothesized early cleavage events genuinely occur within microglial cells.

To explore the strain-like properties of αSyn pathogenesis in the context of proteolytic processing, it is critical to investigate the spectrum of specific αSynΔC variants that can induce different fibril structures via conformational templating and result in dramatically different rates and patterns of propagation [41]. For example, some αSynΔC variants could be potent at enhancing intracellular seeding, while thwarting intercellular propagation. Our arsenal of αSynΔC-specific antibodies stands as a valuable tool in the synucleinopathies research landscape. Indeed, future investigations employing these antibodies for post-mortem analysis in human synucleinopathies should explore the extent to which glial αSynΔC pathology might have been underappreciated by conventionally used antibodies. These investigations have the potential to unveil an αSynΔC proteolytic fingerprint, delineating distinct truncation site patterns associated with each disease. This, in turn, paves the way for the development of differentiating biomarkers for various synucleinopathies. Crucially, pinpointing the precise truncation sites linked to disease mechanisms opens avenues for therapeutic exploration targeting enzymes cleaving at these residues. This potential modification of toxic species levels holds promise for developing disease-ameliorating therapeutics.

### Supplementary Information

Figure S1. Paucity of inclusions in the CNS of TgM83^+/-^ and TgM20^+/-^ injected with control human brain lysates. Representative images of immunohistochemical staining within CNS tissue from (a) TgM83^+/-^ and (b) TgM20^+/-^ mice injected with human control brain previously described [21]. Immunohistochemistry was performed with antibodies 3H19 (αSyn 110–119), 2G5 (αSynΔC103), 1A2 (αSynΔC114), 10A4 (αSynΔC122), 5C1 (αSynΔC125) or 2G7 (αSynΔC129). Selected brain regions, including HPF (hippocampal formation), SUB (subiculum), MY (medulla), and SP (spine) are depicted. Scale bar: 100 µm. Figure S2. Similar distribution profile of αSyn pathology and carboxy-truncations thereof induced by lysates from both MSA cases. Semi-quantification comparing the regional distribution and burden of 3H19 positive inclusions to αSynΔC-positive pathology after inoculation with MSA case 1 (a, c) or MSA case 2 (b, d) in TgM83^+/-^ (a, b) and TgM20^+/-^ mice (c, d).

## Data Availability

The datasets used and analyzed during the current study are available from the corresponding author upon reasonable request.
