# Supplementary figures and images for: A multiverse of α-synuclein: investigation of prion strain properties with carboxyl-terminal truncation specific antibodies in animal models
Source: Acta Neuropathol Commun. 2024 Jun 10;12:91. doi: 10.1186/s40478-024-01805-z (PMC11163735; doi:10.1186/s40478-024-01805-z)

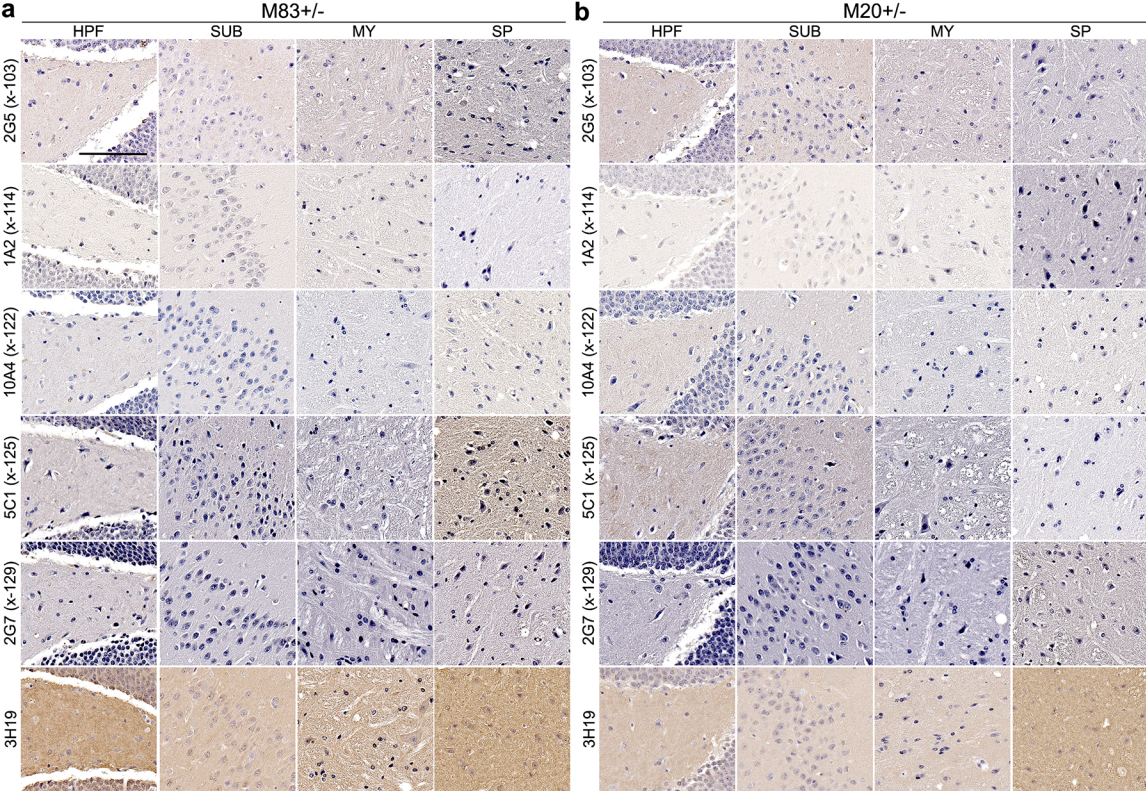

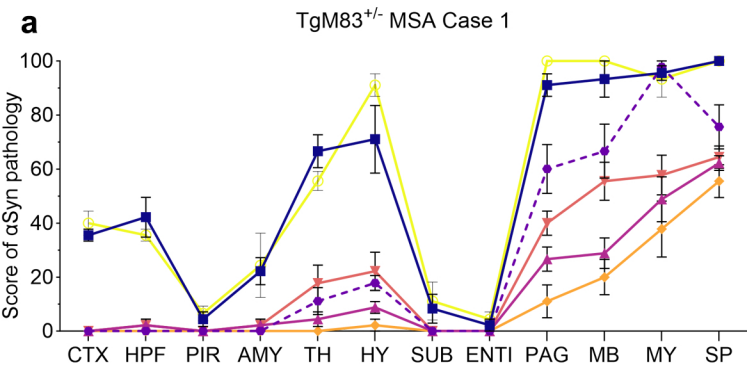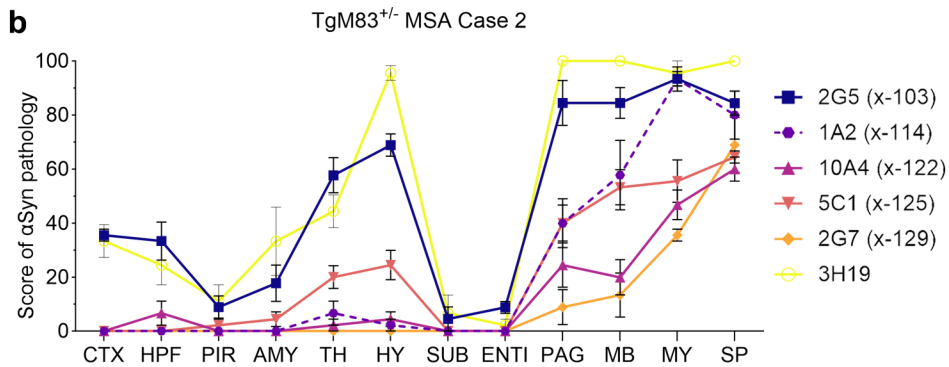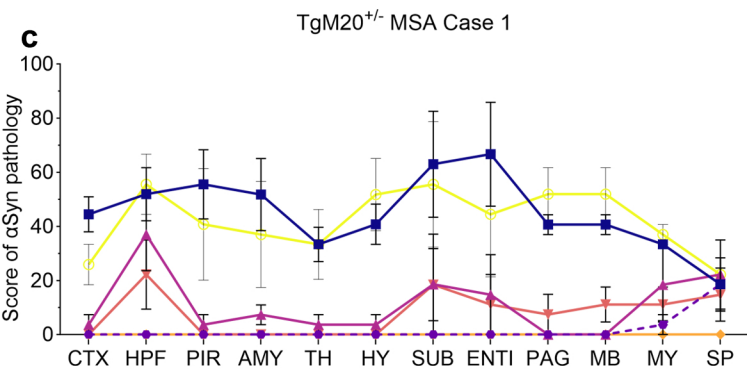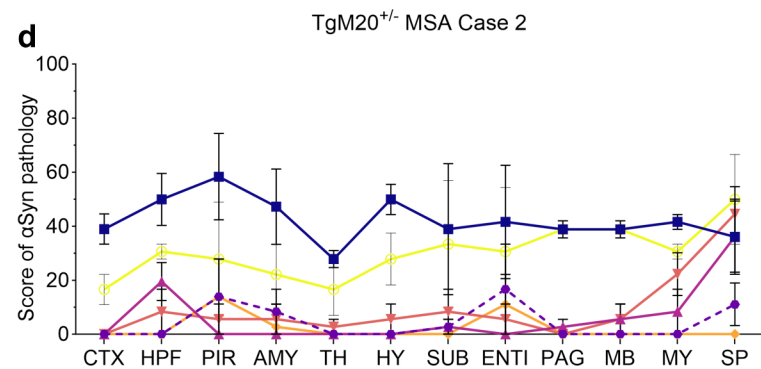

**Supplementary Figure 2**

Supplement: Supplementary file 1 — Figure S1. Paucity of inclusions in the CNS of TgM83+/- and TgM20+/- injected with control human brain lysates. Representative images of immunohistochemical staining within CNS tissue from (a) TgM83+/- and (b) TgM20+/- mice injected with human control brain previously described [21]. Immunohistochemistry was performed with antibodies 3H19 (αSyn 110–119), 2G5 (αSynΔC103), 1A2 (αSynΔC114), 10A4 (αSynΔC122), 5C1 (αSynΔC125) or 2G7 (αSynΔC129). Selected brain regions, including HPF (hippocampal formation), SUB (subiculum), MY (medulla), and SP (spine) are depicted. Scale bar: 100 µm. Figure S2. Similar distribution profile of αSyn pathology and carboxy-truncations thereof induced by lysates from both MSA cases. Semi-quantification comparing the regional distribution and burden of 3H19 positive inclusions to αSynΔC-positive pathology after inoculation with MSA case 1 (a, c) or MSA case 2 (b, d) in TgM83+/- (a, b) and TgM20+/- mice (c, d). [file 40478_2024_1805_MOESM1_ESM.pdf]
